# Reliability of a clinical sensory test battery in patients with spine-related leg and arm pain

**DOI:** 10.1002/ejp.2267

**Published:** 2024-03-25

**Authors:** C. Bender, S. Karstens, F. Muth, G. Baskozos, A.B. Schmid

**Affiliations:** 1 Zurich University of Applied Sciences, School of Health Professions, Institute of Physiotherapy, Winterthur, Switzerland; 2 Division of Therapeutic Sciences, Department of Computer Science, Trier University of Applied Sciences, Trier, Germany; 3 MEDIAN Vesalius-Klinik, Bad Rappenau, Germany; 4 Nuffield Department of Clinical Neurosciences, University of Oxford, Oxford, United Kingdom

## Abstract

**Background:**

The current standard to evaluate the presence of somatosensory dysfunctions is quantitative sensory testing, but its clinical utility remains limited. Low-cost and time-efficient clinical sensory testing (CST) batteries have thus been developed. Recent studies show moderate to substantial reliability in populations with neuropathic pain. This study evaluates the inter- and intra-tester reliability in people with spine-related leg and arm pain, representing mixed pain mechanisms.

**Methods:**

Fifty-three patients with spine-related leg (n = 41) and arm pain (n = 12) attended three CST sessions. The CST battery consisted of eleven tests, determining loss and gain of sensory nerve function. CST was performed by the same investigator twice and by an additional investigator to determine inter- and intra-tester reliability. Fleiss’ (inter-tester) and Cohen’s (intra-tester) kappa were calculated for dichotomised and intraclass correlation coefficients (ICC) for continuous outcomes.

**Results:**

Fleiss’ kappa varied among modalities from fair to substantial (K= 0.23 to 0.66). Cold, warm, and vibration detection thresholds, and cold and pressure pain thresholds reached kappa > 0.4 (moderate to substantial reliability). Cohen’s kappa ranged from moderate to substantial (K= 0.45 to 0.66). Reliability of windup ratio was poor (ICC < 0.18).

**Conclusion:**

CST modalities with moderate to substantial inter-tester reliability could be of benefit as a screening tool. The moderate to substantial intra-tester reliability for all sensory modalities (except windup ratio) supports their potential use in clinical practice and research to monitor somatosensory changes over time in patients with spine-related limb pain of mixed pain mechanisms.

## Introduction

Somatosensory dysfunction is a hallmark feature of neuropathic pain (Colloca et al., 2017), but may also occur in nociceptive pain conditions (Moloney et al., 2015; Tampin et al., 2012). Clinically, these can present as loss (e.g. hypoesthesia or hypoalgesia) or gain (e.g. allodynia or hyperalgesia) of sensory function (Baron et al., 2017). The current reference standard to evaluate the presence and nature of somatosensory dysfunction is quantitative sensory testing (QST). QST is a standardised method to quantify and monitor both loss and gain of nerve function by evaluating somatosensory modalities mediated by primary sensory afferents (Rolke et al., 2006). Promisingly, some QST modalities might have prognostic value for the development of musculoskeletal pain and disability (Georgopoulos et al., 2019; Petersen et al., 2021), highlighting the potential benefits of monitoring specific somatosensory modalities in clinics. While QST is considered a valid measurement to identify somatosensory dysfunction (Rolke et al., 2006), the equipment is expensive, requires training, and is time-consuming, thus limiting its clinical application.

To overcome these barriers, research effort has gone into the development of clinical sensory test (CST) batteries to detect somatosensory dysfunctions with low-cost equipment and higher time-efficiency (Reimer et al., 2020; Ridehalgh et al., 2018; Zhu et al., 2019a). Several studies have examined the concurrent validity of CST (i.e., correlation with QST) (Koulouris et al., 2020; Reimer et al., 2020; Zhu et al., 2019a). Although concurrent validity varies among the different sensory modalities, thermal and mechanical detection thresholds as well as cold and pressure pain thresholds achieve moderate to relatively strong correlations compared to QST (Zhu et al., 2019). Another aspect adding to the validity of CST batteries is their reliability across different examiners (inter-tester) and by the same examiner on different occasions (intra-tester) (Mokkink et al., 2010). Good reliability of CST is imperative for its use as a screening tool and as a clinical tool to measure outcome and monitor treatment response. Recently, moderate to substantial inter- and intra-tester reliability has been reported for different CST batteries in populations with predominant neuropathic pain (Baad-Hansen et al., 2013; Koulouris et al., 2020; Reimer et al., 2020; Wasan et al., 2020). CST might thus offer benefits compared to a standard neurological examination, especially considering its comprehensive assessment of both loss and gain of function of different fibre populations and the notable variability in inter-tester reliability of routine bedside neurological examinations (Dyck et al., 2010).

It remains unknown whether CST batteries are also reliable in patient populations with mixed pain mechanisms. Spine-related limb pain can involve both neuropathic and nociceptive pain mechanisms, with often subtle somatosensory dysfunction (Attal et al., 2011; Tampin et al., 2012). This study therefore aims to assess the inter- and intra-tester reliability of a CST battery in a population with spine-related leg or arm pain to reflect a wide range of pain mechanisms.

## Methods

### Study Cohort

Patients aged 18 and over experiencing spine-related arm or leg pain were recruited from the Vesalius-Clinic in Bad Rappenau rehabilitation centre and a private physiotherapy practice in Germany. These sites represent secondary and primary care settings respectively and therefore help generalisability of our findings. The clinical diagnosis of unilateral spine-related leg or arm pain was made by physiotherapists specialised in musculoskeletal disorders. As there is no diagnostic gold standard to identify spinal related leg and arm pain, we relied on a pragmatic approach based on a detailed subjective and objective assessment. The following two criteria had to be fulfilled: (1) Pain radiating below the gluteal fold in case of spine-related leg pain (Lin et al., 2013); and below the acromion in case of spine-related arm pain, and (2) the limb pain had to be modifiable through spinal movement (Rastogi et al., 2022). Patients included those with painful radiculopathy, radicular pain or spine-related somatic referred pain. Patients were excluded if there was evidence of a central nervous system disorder or metabolic conditions (e.g., myelopathy or diabetes), a clinical diagnosis of anxiety or depression (indicated by a HADS-D total score of > 21 (Mitchell et al., 2010; Vodermaier and Millman, 2011)) as well as bilateral spine-related limb pain. Patients with insufficient command of the German language to complete questionnaires and follow instructions were excluded. The study protocol was approved by the ethics committee from the department of computer science at the Trier University of Applied Sciences (03-2021). Prior to study entry, all participants gave their informed written consent. The reporting of the study is consistent with the guideline for reporting reliability and agreement studies (Kottner et al., 2011).

### Study Procedure

**Figure 1:**
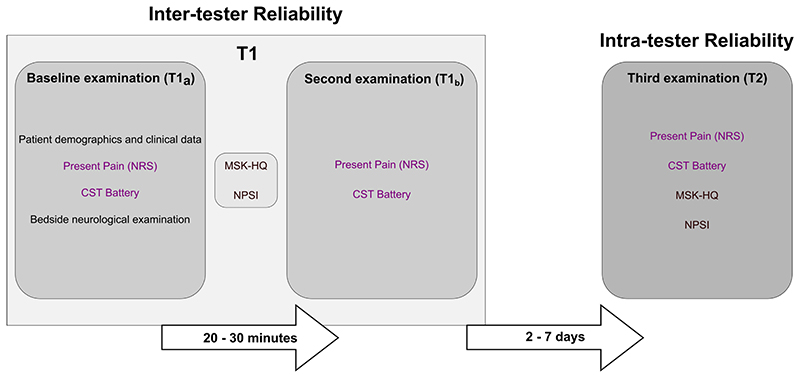
Study protocol. CST, Clinical Sensory Testing; NPSI, Neuropathic Pain Symptom Inventory; NRS, Numeric Rating Scale; MSK-HQ, Musculoskeletal Health Questionnaire

Participants attended three examinations: two on the same day (T1_a_ and T1_b_) and the third after a period of two to seven days (T2) (Fig. 1). During the first examination, demographics and clinical data including age, pain duration, MRI evidence of nerve root compression if available, and findings of a bedside neurological examination (Appendix S1) were recorded by the main investigator (CB). Based on the information available for each patient, we evaluated the presence of probable and definite neuropathic pain according to the grading system by Finnerup et al. (2016). Patients were asked to define their area of maximal pain, and this was marked by one of the examiners on a body diagram. Patients rated the present pain intensity on an 11-point numeric rating scale (NRS, 0 = no pain; 10 = the worst pain imaginable) at the start of the three examinations.

The CST battery was conducted by three physiotherapists specialised in musculoskeletal therapy who had at least five years of professional experience. Before data collection, the main investigator (CB) was trained online for one hour in the CST performance including wording and rating criteria by a specialist who was involved in the original design of the CST (AS). The main investigator then provided an in-person training session to the other two examiners for standardisation purposes. Four months after the start of data collection, the main investigator organised a follow-up training with the other two examiners to prevent drifts in performance.

To determine inter-tester reliability, the first CST session (T1_a_) was performed by one examiner, followed by the second session (T1_b_) by a second examiner twenty to thirty minutes later. The main investigator (CB) was always involved as one examiner in T1_a_ or T1_b_. The examiners were staff from each of the two institutions. We have not standardised or randomised the order of examiners between T1_a_ and T1_b_ due to logistical issues with therapist availability. In case of a pain flare up after T1_a_ (defined by an increase of pain intensity ≥ 2 points on the NRS), the start of T1_b_ was delayed until the pain decreased to levels recorded at T1_a_.

To determine intra-tester reliability, participants returned to the study site for a third CST assessment (T2) within two to seven days after their first session. The main investigator (CB) performed the CST at T2 in all participants.

Examiners were blinded to the CST results of the other examiners but blinding of the patients and same examiner between T1 and T2 was not possible. However, the CST battery contains eleven tests and we considered it highly unlikely that the examiner or patients would remember outcomes. Throughout all three sessions, examiners were blinded to the outcomes of the questionnaires.

### CST battery

**Figure 2:**
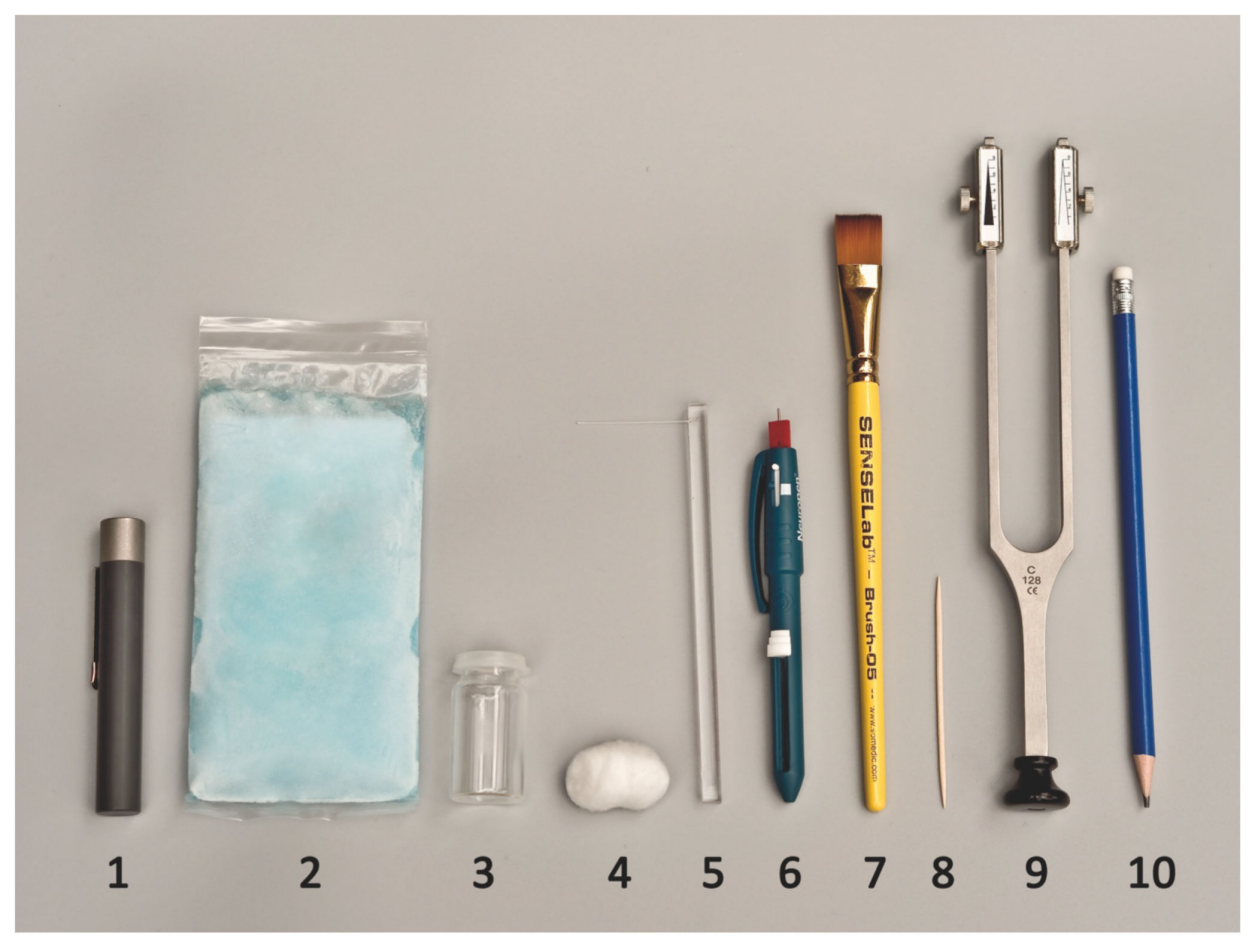
CST devices used in the testing protocol. (1) TipTherm© for cold and warm detection threshold, (2) cooling pack for cold pain threshold, (3) glass vial for heat pain threshold, (4) cotton wool for mechanical detection threshold, (5) von Frey filament 256mN and (6) Neurotip (Neurpen©, Owen Mumford) for mechanical pain threshold, (7) brush (SESELab™-Brush-05) for dynamic mechanical allodynia, (8) toothpick for wind-up ratio, (9) tuning fork 128 Hz for vibration detection threshold, (10) eraser for pressure pain threshold; CST, Clinical Sensory Testing

The CST battery was based on previously published testing protocols (Ridehalgh et al., 2018; Scholz et al., 2009; Zhu et al., 2019). It has been validated as a screening tool to detect sensory dysfunction by identifying deviations from normal levels (i.e., beyond one or two standard deviations of QST z-scores from healthy controls) using dichotomous scaling (normal/abnormal) in individual patients (Zhu et al., 2019). The protocol consisted of eleven readily available and easy-to-use devices determining loss and gain of sensory nerve function (Fig. 2). Before performing the CST, the examiner determined skin temperature in the area of maximal pain using an infrared thermometer (TFA® Infrared-thermometer FLASH PEN). The required skin temperature was set to at least 31°C. Warm packs were used to reach target temperature if required. The order of the CST battery was standardised. After completion of one test modality, we moved immediately to the next test with no breaks between the modalities to accommodate time-constraints imposed by a clinical setting. The test protocol took about ten to fifteen minutes and was based on two time measurements taken during examiner training with patients in both primary and secondary care settings. Participants were tested with eyes closed first on the symptom-free contralateral control area, followed by the corresponding area of maximal pain. The area of maximal pain marked on the body diagram was available to the examiners at each session.

#### Modalities for loss of function

First, a TipTherm© was used to determine the ability to discriminate thermal sensation. For cold detection threshold (CDT), the metal end of the TipTherm© was used while warm detection threshold (WDT) was tested with the polymer end, which is generally perceived as warm or neutral at room temperature. Both modalities were tested for 2 s on the patients’ skin.

Mechanical detection threshold (MDT) was tested via a light stroke of 2-3cm and 1 sec duration using a ball of cotton wool.

Vibration detection threshold (VDT) was evaluated with a tuning fork (Rydel-Seiffer, Arno Barthelmes & Co. GmbH Germany) of 128 Hz frequency which was placed for 10 sec on the skin. The Amplitude of the tuning fork was standardised by releasing the metal fork from a fully approximated position.

Mechanical pain threshold (MPT) was evaluated in two ways. First with a standardised von Frey filament weighing 256mN (MPT VF256 loss of function (LoF)) and second with a pinprick (MPT PP) using a Neurotip mounted on a Neuropen© (Owen Mumford). For the latter, the stimulus was standardised using the integrated spring pressure of 40g. Both modalities were placed for 1-2 s on the patients’ skin.

#### Modalities for gain of function

For cold pain threshold (CPT), a cooling pack (8cm x 15cm) previously stored in a freezer compartment was placed for 10 sec on the patients’ skin.

Heat pain threshold (HPT) was evaluated with a glass vial filled with hot water (40°C) and placed for 10 sec over the skin. The water was heated to 40°C with a kettle and temperature determined with the laser thermometer.

MPT was performed as above with a standardised von Frey filament weighting 256mN but recorded as gain of function (MPT VF256 gain of function (GoF)) if perception was increased on the maximal pain site compared to the contralateral site. Pressure pain threshold (PPT) was evaluated with an eraser mounted on a pencil. Pressure was applied for 10 s over the closest muscle belly to the area of maximal pain. The pressure was sufficient to indent the soft tissue leading to skin blanching.

Wind-up Ratio (WUR) was established with a toothpick by applying a single stimulus followed by a train of 10 stimuli at a frequency of 1/sec. Participants rated the pain on a 11-point NRS from 0 (no pain at all) to 10 (worst pain imaginable). WUR was calculated as the ratio of the single stimulus rating over the average rating for the train of stimuli. In a post hoc analysis, we also calculated the difference between the single and train of stimuli to avoid data loss due to zero ratings on the single stimulus. Dynamic mechanical allodynia (DMA) was assessed with a brush (SESELab™-Brush-05, Somedic Sweden) by gently stroking the skin three times over a length of 2-3cm with a 1 sec duration.

#### CST Interpretation

Immediately after the application of each CST stimulus, patients were asked whether the stimulus applied over the affected site was perceived as increased, decreased or the same intensity compared to the contralateral control area. In loss of function modalities (detection thresholds, MPT PP and MPT VF256) a perception of decreased sensation was interpreted as loss of function. In the gain of function modalities (pain thresholds), a perception of increased sensation was considered to reflect gain of function. DMA was rated as present (pain provocation) or absent (no pain provocation).

### Questionnaires

Participants completed the Musculoskeletal Health Questionnaire (MSK-HQ) and Neuropathic Pain Symptom Inventory (NPSI) at both T1 and T2. These questionnaires served the primary purpose of evaluating symptom stability, checking that participants’ characteristics remained consistent throughout the testing period.

This stability assessment was crucial to then establish the intra-tester reliability of CST modalities. To reduce potential information bias, the baseline questionnaires were completed between T1_a_ and T1_b_ to shift participants’ focus off the sensory testing.

#### Musculoskeletal Health Questionnaire - MSK-HQ

The German translation of the MSK-HQ (MSK-HQ_G_) was implemented to assess patient health status on a functional level. With 14 items on pain, disability, emotions, sleep, and self-confidence to manage the condition, the MSK-HQ provides a holistic view of the impact of the condition (Hill et al., 2016; Karstens et al. 2021). The MSK-HQ is scored from 0-56, with a higher score indicating better musculoskeletal health status. The minimal important change has been estimated in a range of musculoskeletal conditions as 8.5 points (Karstens et al., 2021). The German translation shows good test-retest reliability, and good construct validity (Karstens et al., 2021).

#### Neuropathic Pain Symptom Inventory - NPSI

The German version of the NPSI (NPSI-G) was used to obtain information about different pain characteristics. The NPSI includes five cluster of items (superficial spontaneous pain, deep spontaneous pain, paroxysmal pain, evoked pain, as well as paraesthesia and dysaesthesia) to discriminate and quantify different dimensions of neuropathic pain. In the NPSI, each sub score can range from 0-10 and the total score from 0-100 with higher scores representing higher symptom severity (Bouhassira et al., 2004). It has been showed that the NPSI is sensitive to detect change (Bouhassira et al., 2004; Sommer et al., 2011).

### Statistical analysis and sample size

Before statistical analysis was performed, the CST variables (increased, decreased, or normal) were dichotomised (normal or abnormal), as validated previously by Zhu et al. (2019). The dichotomous scaling offers clinicians the advantage of quickly identifying the presence or absence of somatosensory dysfunction in an individual patient. A perceived decreased response (for loss of function tests) or increased response (for gain of function tests) compared to the control area was defined as a sensory dysfunction (abnormal). An equal response was defined as normal function (normal).

For dichotomised measures, inter-tester agreement (T1_a_ and T1_b_) was estimated utilising Fleiss’ Kappa (accommodates non fully cross-over designs) while intra-tester reliability (T1 and T2) was estimated using Cohen’s Kappa statistics. Inter-tester and intra-tester agreement estimations were interpreted adopting the method introduced by Landis and Koch ((Landis and Koch, 1977): < 0 poor, 0 − 0.2 slight, 0.21 − 0.4 fair, 0.41 − 0.6 moderate, 0.61 − 0.8 substantial, 0.81 − 1.0 almost perfect agreement). We also provide observed agreement and proportion of specific agreement for each test as a measure to assist the assessment of agreement of individuals in clinical practice.

Symptom stability from T1 to T2 was measured based on the change of the NPSI and MSK-HQ for all dichotomised sensory modalities. We applied a Shapiro-Wilk test to test for normal distribution, followed by a paired t-test or Wilcoxon test to determine if the scores of the two questionnaires changed between timepoints. Due to statistically significant changes in the questionnaire scores over time, which may have impacted on the CST findings and thus reliability, a two stage logistic regression model for analysing the intra-tester agreement with the change in scores as covariates was performed as a post hoc sensitivity analysis to check if the agreement of the two testers was still better than chance, even when taking both questionnaire scores as covariates into account (Lipsitz et al., 2003). We considered the two-stage regression as an extension of Kappa that can handle covariates. In the first regression stage the marginal probabilities for each one of the testers to give an abnormal rating, given the covariates, were calculated. Then an offset term, representing agreement by chance only, was formulated as the logit of agreement between the fitted probabilities for each one of the testers to give an abnormal rating. This offset was then used alongside the covariates (change in MSK-HQ and NPSI from T1 to T2) in the second logistic regression stage to model the overall, chance corrected, agreement between the two testers. This method first used the random agreement of the two testers (first stage - offset) as well as the chance-corrected agreement of the covariates and an offset (second stage). We then tested the hypothesis that the overall chance-corrected agreement considering the covariates was higher than agreement due to chance only using a one-sided t-test. For the continuous measures (WUR and temporal summation of pain (TSP)), Intraclass correlation coefficients (ICC) were calculated to determine the magnitude of inter-tester and intra-tester agreement. We computed inter-tester ICC based on a one-way random-effects model, single measures, and absolute-agreement of three observers. Intra-tester ICC was calculated utilising a two-way mixed-effects model, single measures, and absolute-agreement. Due to the high amount of zero ratings during the first stimulus of WUR testing, we performed a post hoc ICC analysis for TSP based on the difference between the average rating for the train of stimuli and the single stimulus rating. ICC was interpreted under the random according to Koo and Li: ICC < 0.5 poor, ICC > 0.5 moderate, ICC > 0.75 good, and ICC > 0.9 excellent correlation (Koo and Li, 2016).

All statistical analyses were performed using the statistical computing language R (v4.0.0; R Core Team 2020). Sample size estimation was performed a priori by applying a confidence interval approach (Rotondi and Donner, 2012). The desired bounds for a two-sided confidence interval were set to +/- 0.2 from the anticipated preliminary kappa value of 0.61, resulting in a sample size estimation of a minimum of 53 participants.

## Results

Fifty-three patients were recruited for the study procedure and all but one completed all three sessions (Covid-related dropout at T2). Patient characteristics are described in Table 1. Forty-one patients had spine-related leg pain and 12 spine-related arm pain. MRI data were available for 29 patients. A neurological deficit upon bedside neurological examination was found in 30 patients. Eleven patients (20.8%) met the criteria for probable neuropathic pain, while nineteen patients (35.9%) were identified to have definite neuropathic pain (Finnerup et al., 2016). We recorded a decrease in present pain NRS from 4 (SD = 2.4) at T1 to 3.3 (SD = 2.4) at T2. A pain flare-up between T1_a_ and T1_b_ was recorded in three patients. Their second testing session was delayed until the pain returned to baseline values. Details on symptom stability between T1 and T2 according to the MSK-HQ and NPSI can be found in Table S2. We did not consider DMA in our statistical analysis as it was rated as absent in all patients.

### Inter-tester reliability

The percentage of observed agreement for dichotomised CST modalities ranged from 66% (MPT PP) to 83% (CDT), with CDT, CPT, and PPT reaching > 80% agreement (Table 2). Kappa statistics for dichotomised modalities ranged from fair (0.23 for MPT VF256 (GoF)) to substantial (0.66 for CDT). We identified fair agreement for MDT, MPT (both loss and gain of function), MPT PP, and HPT; moderate agreement for WDT, VDT, and CPT; and substantial agreement for CDT and PPT. We were able to estimate WUR only for seventeen patients as thirty-six did not experience pain on the initial stimulus. ICC for WUR was poor between examiners (0.14) (Table 3).

### Intra-tester reliability

The mean time interval between T1 and T2 was 3.7 days. The percentage of observed agreement for dichotomised CST modalities ranged from 73% (MPT VF256 (GoF)) to 85% (CPT), with CDT, MDT, CPT, and HPT reaching > 80% agreement (Table 4). Kappa statistics for dichotomised modalities ranged from moderate (0.45 for VDT and MPT VF256 (GoF)) to substantial (0.66 for CPT). We identified moderate agreement for all modalities, except for CDT and CPT which reached substantial agreement (Table 4).

The changes of the MSK-HQ and NPSI were found to be statistically significant from T1 to T2 (Table S2). Therefore, a two-stage logistic regression analysis for intra-tester agreement adjusted for the two covariates NPSI and MSK-HQ followed by a one-sided t-test revealed that the agreement of the testers could not be explained by chance only, i.e. agreement between fitted probabilities of each tester modelled after the covariates alone (Table 5). ICC for WUR, was poor for intra-tester reliability (0.18) (Table 3). The post hoc ICC analysis for TSP revealed comparably poor inter-tester and intra-tester reliability (Table S3).

Further details on proportion of specific agreement relating to whether sensory modalities were rated as abnormal or normal can be found in Table S1.

## Discussion

This study investigated the inter-tester and intra-tester reliability of a comprehensive, time-efficient, and low-cost CST battery in a sample of patients with spine-related leg and arm pain. We found that most CST modalities achieved moderate reliability (inter-tester: WDT, VDT, and CPT; intra-tester: WDT, MDT, MPT VF256 (LoF), MPT PP, VDT, HPT, MPT VF 256 (GoF), and PPT) while four modalities reached substantial agreement (inter-tester CDT and PPT; intra-tester: CDT and CPT). We found poor inter- and intra-tester agreement for WUR as well as fair inter-tester agreement for five CST modalities (MDT, MPT (LoF), MPT PP, HPT, and MPT (GoF)) which challenges their value in this patient population.

### Inter-tester reliability

A clinical test must have sufficient inter-tester reliability to be valid as a screening tool in clinical practice. Overall, inter-tester reliability ranged substantially between sensory modalities. We found that three sensory modalities (WDT, VDT, and CPT) reached comparable and two (CDT and PPT) achieved better inter-tester reliability than previously reported in similar CST batteries among patients with neuropathic pain (Reimer et al., 2020; Wasan et al., 2020). Notably, this is the first study to investigate the reliability of thermal thresholds using the TipTherm® device. Arguably, the TipTherm® might be more time-efficient than other devices to screen for thermal detection thresholds as it does not need time to adjust the target temperature. Five sensory modalities (MDT, MPT PP, MPT VF256 (LoF and GoF), HPT, and WUR) were found to have worse inter-tester reliability than previously reported (Schmid et al., 2009; Vroomen et al., 2000; Wasan et al., 2020). Several reasons may have led to this divergence. First, Schmid et al (2019). examined MDT exclusively in the upper extremity, which has increased tactile sensitivity compared to the lower limb (Ackerley et al., 2014). Second, more rigorous training, including a certification exam might add to higher inter-tester reliability found in Wasan et al. (Wasan et al., 2020). Third, we used lower temperatures to detect HPT compared to other studies (Reimer et al., 2020; Wasan et al., 2020). Potentially, slightly higher temperatures of 45°C to 47°C create stronger heat hyperalgesia and therefore achieve higher inter-tester reliability (0.62 and 0.55, respectively) than more subtle heat (40°C) used here. Promisingly, we found moderate to substantial inter-tester reliability for several sensory modalities (WDT, VDT, CPT, CDT, and PPT), which further supports their usefulness as screening devices to detect somatosensory dysfunction in populations with mixed pain mechanisms. However, poor to fair inter-tester reliability was found for MDT, MPT PP, MPT 256VF (LoF and GoF), HPT, and WUR, which questions their clinical value.

### Intra-tester reliability

Good intra-tester reliability is important to monitor changes over time. Except for WUR, all CST modalities showed moderate to substantial intra-tester reliability. These levels of agreement are in accordance with previous research (Koulouris et al., 2020; Wasan et al., 2020). However, we found poorer reliability for WUR than previously reported (Nothnagel et al., 2017; Schmid et al., 2019). One possible explanation is that we included a patient population whereas previous research was performed in healthy participants which are expected to show more homogenous sensory function. Of note, our analysis for WUR was underpowered as we had to exclude a high number of participants from WUR analysis (n = 36 of 53 for inter-tester and n = 29 of 52 for intra-tester reliability) due to zero ratings on the single stimulus. Alternative analysis of TSP using all participants by calculating differences of ratings did not improve reliability. The toothpick we used for WUR was not sharp enough to elicit a painful response in every participant. In line with this, our previous work suggested that a toothpick is inferior to a Neurotip in identifying small-fibre degeneration (Ridehalgh et al., 2018).

While we did identify a statistically significant improvement of NPSI and MSK-HQ scores from T1 to T2, clinically, NPSI change was very small (2.88 out of 100) and MSK-HQ change (2.57 out 56) was below the minimal important change (8.5) (Karstens et al., 2021) and the agreement of the testers could not be explained by these two covariates alone. This sensitivity analysis further lends confidence in our intra-tester reliability results.

Taken together, the results of this study show moderate to substantial intra-tester reliability except for WUR, supporting the usage of most CST modalities to monitor change of somatosensory function in clinical practice and research in populations with spine-related limb pain of mixed pain mechanisms.

## Limitations and further directions

Several limitations need to be considered. Although we included the pre-specified sample size of 53 participants, one participant missed the third testing session and we found spine-related arm pain underrepresented (n = 12) compared to spine-related leg pain (n = 41). This limits the generalizability of our results in this population and also increased the degree of uncertainty regarding the true reliability values of the measurements for spine-related arm pain. Therefore, it may be necessary to conduct further research with a larger sample size to obtain more robust reliability estimates specifically for spine-related arm pain.

In addition, we have opted to dichotomise the outcomes for all CST parameters except WUR. Whereas this is useful as a screening tool, this limits the CST’s ability to quantify the degree of sensory dysfunction which would be useful for comparison between patients, or within patients over time. Optimally CST modalities would need to allow quantification (e.g. NRS for pain to quantify gain of function measurements or utilizing a tuning fork with a scale) as has been implemented in other CST batteries (Koulouris et al., 2020; Reimer et al., 2020).

Blinding of the participants and examiners from T1 to T2 could not be achieved, possibly introducing recall bias and impacting the results of intra-tester reliability. Given the high number of tests, we consider it unlikely that participants or investigators could remember each rating. Future studies could include checks for blinding for instance by asking the examiner before T2 to guess the outcome of the sensory tests combined with the certainty of the guess (Kolahi et al., 2011). In addition, recall bias could be reduced using a longer time-interval between T1 and T2. However, given that our patient population received intensive physiotherapy management and indeed improved over the short testing period, we did not consider it appropriate to extend the time interval between the testing sessions.

Further studies are required to investigate whether CST modalities are sensitive to change as well as their possible prognostic ability. Notably, it has been shown that QST modalities like temporal summation and thermal pain detection thresholds have moderate correlation to pain and disability outcomes respectively (Georgopoulos et al., 2019). In future research, it will be valuable to determine whether CST modalities have prognostic abilities comparable to those of QST. This is particularly relevant as CST is more clinically feasible. Several studies have investigated different aspects of validity and reliability of different CST batteries and synthesizing them (e.g., meta-analysis) will help to determine the optimal CTS battery and estimate their value as a cost-effective alternative for QST in different populations.

## Conclusion

This study shows that the inter-tester reliability of a low-cost and time-efficient CST battery varies from poor to substantial depending on the sensory modality. This variability in inter-tester reliability suggests that only some modalities are likely to be useful as screening tools. In contrast, the CST battery has moderate to substantial intra-tester reliability, supporting its potential application in clinical practice and research to monitor different sensory modalities in this patient population.

## Supplementary Material

Supplementary Materials

## Figures and Tables

**Table 1:** Patient characteristics

	N = 53^a^
Age [mean ± SD] (range)	52.4 ± 10.3 (31-80)
Sex [n] (%)	
female	31 (58%)
male	22 (42%)
Area of maximal pain [n] (%)	
Upper limb	12 (22.6)%
Upper arm	1 (1.9%)
Forearm	5 (9.4%)
Hand	6 (11.3%)
Lower limb	41 (77.4%)
Thigh	19 (35.9%)
Lower leg	15 (28.3%)
Foot	7 (13.2%)
Pain duration, months [mean ± SD] (range)	23.0 ± 54.4 (1-360)
Present pain intensity NRS [mean ± SD]	
T1_a_	4.0 (2.4)
T1_b_	3.9 (2.4)
T2	3.3 (2.4)
MSK-HQG score [mean ± SD]	
T1	33.0 ± 11.1
T2 (n = 52)	34.8 ± 10.2
NPSI-G score [mean ± SD]	
T1	28.9 ± 18.2
T2 (n = 52)	26.1 ± 18.1
MRI evidence of nerve root compression [n] (%)	
Cervical spine	10 (18.9%)
C4/5	2 (3.8%)
C5/6	4 (7.6%)
C6/7	4 (7.6%)
Lumbar spine	21 (39.6%)
L3/4	3 (5.7%)
L4/5	10 (18.9%)
L5/S1	9 (17.0%)
Loss of function in bedside neurological examination [n] (%)	
Cervical spine	
Sensory (light touch)	6 (11.3%)
Muscle strength	4 (7.6%)
Tendon reflex	4 (7.6%)
Lumbar spine	
Sensory (light touch)	15 (28.3%)
Muscle strength	13 (24.5%)
Tendon reflex	7 (13.2%)
Neuropathic pain grading [n] (%)	
Probable	11 (20.8%)
Definite	19 (35.9%)

a unless noted differently; MSK-HQ, musculoskeletal health questionnaire; NPSI, neuropathic pain symptom inventory; NRS, numeric rating scale; SD, standard deviation

**Table 2:** Observed agreement and inter-tester reliability (T1a and T1b) for dichotomised CST modalities (n = 53)

Modalities	Observed Agreement (%)	Inter-tester Reliability (k)	Lower and upper limits of CI (95%) for k
**Loss of function**			
CDT	83%	0.66	0.43 ; 0.85
WDT	79%	0.56	0.28 ; 0.78
MDT	74%	0.40	0.12 ; 0.65
MPT VF265 (LoF)	62%	0.24	-0.05 ; 0.49
MPT PP	66%	0.32	0.06 ; 0.56
VDT	79%	0.46	0.13 ; 0.73
**Gain of function**			
CPT	81%	0.60	0.37 ; 0.80
HPT	75%	0.39	0.06 ; 0.66
MPT VF265			
(GoF)	68%	0.23	-0.11 ; 0.52
PPT	81%	0.61	0.38 ; 0.81

0.82 − 1, almost perfect (dark green); 0.61 − 0.8, substantial (light green); 0.41 − 0.6, moderate (yellow); 0.21 − 0.4, fair (orange); 0 − 0.2, slight (red); < 0, poor (grey); CDT, cold detection threshold; CPT, cold pain threshold; CST, clinical sensory testing; HPT, heat pain threshold; MDT, mechanical detection threshold; MPT VF265, mechanical pain threshold van Frey hair weighting 265 mN; LoF/GoF, loss/gain of function; MPT PP, mechanical pain threshold pin prick; PPT, pressure pain threshold; VDT, vibration detection threshold; WDT, warm detection threshold

**Table 3:** Inter-tester (n = 17) and intra-tester reliability (n = 23) for WUR

Modalities	Inter-tester Reliability (ICC)	Lower and upper limits of CI (95%) for ICC	Intra-tester Reliability (ICC)	Lower and upper limits of CI (95%) for ICC
WUR	0.14	0 − 0.46	0.18	-0.19 ; 0.52

>0.9, excellent (green); >0.75, good (light blue); >0.5, moderate (blue); < 0.5, poor (pink); ICC, intraclass correlation coefficient; WUR, wind-up ratio

**Table 4:** Observed agreement and intra-tester reliability (T1 and T2) for dichotomised CST modalities (n = 52)

Modalities	Observed Agreement (%)	Intra-tester reliability (k)	Lower and upper limits of CI (95%) for (k)
**Loss of function**			
CDT	81%	0.61	0.39 ; 0.83
WDT	77%	0.51	0.27 ; 0.75
MDT	83%	0.59	0.35 ; 0.83
MPT VF265 (LoF)	75%	0.48	0.24 ; 0.73
MPT PP	79%	0.55	0.31 ; 0.79
VDT	79%	0.45	0.16 ; 0.74
**Gain of function**			
CPT	85%	0.66	0.45 ; 0.88
HPT	81%	0.51	0.24 ; 0.79
MPT VF265 (GoF)	73%	0.45	0.20 ; 0.69
PPT	75%	0.50	0.27 ; 0.74

0.82 − 1, almost perfect (dark green); 0.61 − 0.8, substantial (light green); 0.41 − 0.6, moderate (yellow); 0.21 − 0.4, fair (orange); 0 − 0.2, slight (red); < 0, poor (grey); CDT, cold detection threshold; CPT, cold pain threshold; CST, clinical sensory testing; HPT, heat pain threshold; MDT, mechanical detection threshold; MPT VF265, mechanical pain threshold van Frey hair weighting 265 mN; LoF/GoF, loss/gain of function; MPT PP, mechanical pain threshold pinprick; PPT, pressure pain threshold; VDT, vibration detection threshold; WDT, warm detection threshold

**Table 5:** Two stage logistic regression analysis of intra-tester agreement (T1 and T2) adjusted for covariates (MSK-HQ, NPSI)

Modalities	Estimate	t	P value
**Loss of function**			
CDT	1.52	12.01	< 0.005
WDT	1.04	8.33	< 0.005
MDT	1.85	6.56	< 0.005
MPT VF256 (Lof)	1.02	18.10	< 0.005
MPT PP	1.10	24.39	< 0.005
VDT	1.47	7.80	< 0.005
**Gain of function**			
CPT	1.44	40.77	< 0.005
HPT	1.23	7.43	< 0.005
MPT VF256 (GoF)	0.98	20.29	< 0.005
PPT	1.50	9.32	< 0.005

CDT, cold detection threshold; CPT, cold pain threshold; HPT, heat pain threshold; MDT, mechanical detection threshold; MPT VF265, mechanical pain threshold van Frey hair weighting 265 mN; LoF/GoF, loss/gain of function; MPT PP, mechanical pain threshold pinprick; MSK-HQ, musculoskeletal health questionnaire; NPSI, neuropathic pain symptom inventory, PPT, pressure pain threshold; VDT, vibration detection threshold; WDT, warm detection threshold. Results show the hypothesis testing that the chance corrected agreement is equal to the agreement by chance (agreement between the marginal probabilities for each tester to give an abnormal rating by only taking into account the covariates), i.e. their true difference is 0. The column “Estimate” shows the estimate of their true difference, “t” holds the t-statistic and the “P value” shows the significance level of the one-sided t-test P value.

## Significance

We already know that most modalities of clinical sensory test (CST) batteries achieve moderate to substantial inter and intra-tester reliability in populations with neuropathic pain.

This study evaluates the reliability of a CST battery in populations with mixed pain mechanisms. We found inter-tester reliability varied from poor to substantial for sensory modalities questioning the value of some CST modalities. The CST battery showed moderate to substantial intra-tester reliability, suggesting its usefulness to monitor sensory changes over time in this cohort.
