# Supplementary material for: Reliability of a clinical sensory test battery in patients with spine‐related leg and arm pain
Source: Eur J Pain. 2024 Mar 25;28(8):1366–77. doi: 10.1002/ejp.2267 (PMC7616657; doi:10.1002/ejp.2267)
Supplement: Supplementary file 1 — Data S1. [file EJP-28-1366-s001.docx]

**Supplementary material**

**AppendixS1: Clinical-neurological examination**

The clinical-neurological examination included manual muscle testing, somatosensory function, and reflexes.

Manual muscle testing was performed in myotomes C4 to Th1 (spine-related arm pain) and L1 to S1 (spine-related leg pain). All manual muscle testing was performed and graded based on the 5-point British Medical Research Scale from M0 to M5. Muscle power was rated as normal (M5/5) or reduced (M4/5 or less).

Somatosensory function was tested with light touch, using a cotton wool. For the upper limb, the cotton wool was lightly brushed in a circumferential pattern around the upper arm and forearm (starting from C5 dermatome). Each finger was tested separately both from the dorsal and palmar sides. For the lower limb, the cotton wool was also brushed in a circumferential pattern around the upper and lower leg starting from dermatome L2. Each toe was tested separately both from the dorsal and plantar sides. Sensation was rated as normal or decreased.

Reflexes of the biceps (C5-6) and triceps (C6-7) were tested for the upper extremity using a reflex hammer. Patellar tendon reflex (L3-4) and Achilles tendon reflex (S1) were tested for the lower extremity. Reflexes were rated as normal or decreased.

**TableS1: Proportion of specific agreement for all dichotomised modalities**

| **Modalities** | **Inter-tester** | | **Intra-tester** | |
| --- | --- | --- | --- | --- |
|  | Agreement on abnormal (%) | Agreement on normal (%) | Agreement on abnormal (%) | Agreement on normal (%) |
| **Loss of function** |  |  |  |  |
| CDT | 0.84 | 0.88 | 0.77 | 0.83 |
| WDT | 0.73 | 0.83 | 0.70 | 0.81 |
| MDT | 0.56 | 0.81 | 0.71 | 0.88 |
| MPT VF256 (LoF) | 0.58 | 0.66 | 0.67 | 0.80 |
| MPT PP | 0.56 | 0.74 | 0.72 | 0.83 |
| VDT | 0.62 | 0.86 | 0.59 | 0.86 |
| **Gain of function** |  |  |  |  |
| CPT | 0.85 | 0.74 | 0.88 | 0.78 |
| HPT | 0.58 | 0.83 | 0.64 | 0.87 |
| MPT VF256 (GoF) | 0.45 | 0.77 | 0.65 | 0.78 |
| PPT | 0.78 | 0.83 | 0.75 | 0.75 |
| CDT, cold detection threshold; CPT, cold pain threshold; HPT, heat pain threshold; MDT, mechanical detection threshold; MPT VF265, mechanical pain threshold van Frey hair weighting 265 mN; LoF/GoF, loss/gain of function; MPT PP, mechanical pain threshold pinprick; PPT, pressure pain threshold; VDT, vibration detection threshold; WDT, warm detection threshold | | | | |

**TableS2: Symptom stability (according to MSK-HQ, and NPSI) from T1 to T2**

| Self-reported outcomes | Baseline Visit (T1)  [mean ± SD] | Third visit (T2)  [mean ± SD] | P Value |
| --- | --- | --- | --- |
| MSK-HQ^a^ | 33.0 ± 11.1 | 34.8 ± 10.2 | 0.001 |
| NPSI^b^ | 28.9 ± 18.2 | 26.1 ± 18.1 | 0.037 |
| Statistical tests included: ^a^ paired t-test, ^b^ paired Wilcoxon test; MSK-HQ, musculoskeletal health questionnaire; NPSI, neuropathic pain symptom inventory; SD, standard deviation | | | |

**TableS3: Inter-tester (n = 53) and intra-tester reliability (n = 52) for temporal summation of pain (TSP)**

| **Modalities** | **Inter-tester Reliability (ICC)** | **Lower and upper limits of CI (95%) for ICC** | **Intra-tester Reliability (ICC)** | **Lower and upper limits of CI (95%) for ICC** |
| --- | --- | --- | --- | --- |
| TSP | 0.069 | -0.18 – 0.32 | 0.07 | -0.18 ; 0.32 |
| >0.9, excellent (green); >0.75, good (light blue); >0.5, moderate (blue); < 0.5, poor (pink); ICC, intraclass correlation coefficient; TSR, temporal summation of pain | | | | |
